# Factors Associated with Mortality of Lambs Born to Ewe Hoggets

**DOI:** 10.3390/ani12030319

**Published:** 2022-01-28

**Authors:** Anne L. Ridler, Kate J. Flay, Paul R. Kenyon, Hugh T. Blair, Rene A. Corner-Thomas, Emma J. Pettigrew

**Affiliations:** 1 School of Veterinary Sciences, Massey University, Palmerston North 4472, New Zealand; 2 Department of Veterinary Clinical Sciences, City University of Hong Kong, Kowloon, Hong Kong 999077, China; kateflay@cityu.edu.hk; 3 School of Agriculture and Environment, Massey University, Palmerston North 4472, New Zealand; P.R.Kenyon@massey.ac.nz (P.R.K.); H.Blair@massey.ac.nz (H.T.B.); R.Corner@massey.ac.nz (R.A.C.-T.); 4 Wairere Ltd., 835 Wairiri Rd, RD2, Masterton 5882, New Zealand; epettigrew1@gmail.com

**Keywords:** ewe, hogget, lamb, mortality, survival, reproduction, body condition score, dystocia, stillborn, vaginal prolapse

## Abstract

**Simple Summary:**

Pre-weaning lamb deaths limit the production performance from ewes that are bred as hoggets (at 6–9 months of age). The present study aimed to investigate factors that are associated with increased likelihood of lamb death and the cause of that death, in lambs and in ewe hoggets over the lambing period. This was also compared to rates in mature-age ewes and their lambs. Lambs with low birthweights or that were born as twins were more likely to die, as were lambs whose dams had greater live weight changes during pregnancy. Ewes deaths during the lambing period accounted for approximately 11% of lamb deaths. The most common cause of hogget lamb deaths was stillbirth (lamb born dead) but this was an uncommon cause of death in lambs born to mature-age ewes. Management tools to increase lamb birthweights, and supervision of ewe hoggets at lambing time, are recommended.

**Abstract:**

The reproductive performance of ewe hoggets is poorer than that of mature-age ewes due to production of fewer lambs with poorer survival. Scant data are available on the risk factors for, and causes of, the mortality for lambs born to ewe hoggets, the impact of ewe deaths on lamb loss, and the causes of death for lambs born to ewe hoggets vs. mature-age ewes lambing in the same circumstances. In this study, 297 lambs born to 1142 ewe hoggets were necropsied along with 273 lambs born to 1050 mature-age ewes. Low lamb birthweight, multiple litter size, and increasing ewe hogget average daily gain from breeding to late pregnancy were risk factors for lamb mortality. The most common cause of mortality for lambs born to ewe hoggets was stillbirth and the risk factors for stillbirth were similar to those for lamb mortality generally. Approximately 11% of ewe hoggets’ lamb deaths were due to the death of the dam. Causes of mortality differed between lambs born to ewe hoggets vs. those born to mature-age ewes. Management practices to increase ewe hogget lambs’ birthweights (particularly those from multiple litters) and supervision of ewe hoggets at lambing time are recommended.

## 1. Introduction

In seasonal breeds of sheep that lamb once per year, breeding ewe hoggets (6–9 months of age at breeding; approximately 1 year of age at lambing) can be used to increase ewe lifetime productivity and improve farm efficiency [1]. It is well-established that the reproductive performance of ewe hoggets is poorer than that of mature-age ewes and is due largely to the production of fewer lambs with reduced survival [1,2,3,4]. Survival to tailing or weaning in lambs born to ewe hoggets in extensive pastoral livestock systems have been reported to range from 61–89% for singletons and 62–79% for multiple-born lambs [2,5,6,7,8]. 

Most lamb deaths occur within the first three to five days of life and in extensive sheep production systems such as New Zealand and Australia, the most common causes of mortality in lambs born to mature-aged ewes are the starvation-mismothering-exposure (SME) complex and dystocia [9]. However, there is limited published literature regarding causes of perinatal lamb mortality in lambs born to ewe hoggets and a lack of studies investigating potential risk factors or comparing mortality causes for lambs born to ewe hoggets and those born to mature-aged ewes bred to the same rams and lambing at the same time. One New Zealand study reported that the main causes of perinatal mortality in lambs born to ewe hoggets were dystocia followed by SME [5]. In Australia, lamb necropsies from 11 primiparous ewe flocks (seven of which were ewe hogget flocks) attributed 34% of deaths to SME, 24% to dystocia, and 15% to stillbirth [7]. 

A number of lamb-level risk factors for perinatal mortality have been reported. It is well-established that lamb birthweight has a large impact on lamb survival, with both very small and very large lambs being more prone to mortality [9,10,11]. Even accounting for birthweight, male lambs are reported to be at a higher risk [8,11]. At the ewe-level, studies in mature-aged Merino ewes in Australia have demonstrated that maternal nutrition during pregnancy, particularly late pregnancy, was a good predictor of lamb birthweight and survival [12,13]. However, in ewe hoggets, several controlled New Zealand studies showed that differential feeding during pregnancy to achieve varying levels of total liveweight gain did not influence lamb birthweight or survival (reviewed by [1]). In a study undertaken on commercial sheep farms and based on whether or not ewe hoggets were actively lactating in mid-to-late lactation, hoggets at a lower bodyweight prior to lambing, who had poorer weight gain in mid-to late gestation or with poor BCS prior to lambing, were less likely to rear a lamb to weaning compared with heavier, faster-growing or better BCS hoggets [6]. However, in that study neither the timing nor causes of lamb deaths were investigated. 

It is of interest to note that in the majority of studies investigating perinatal lamb mortality, the contribution of ewe death to lamb wastage has not been reported, although one New Zealand study reported that at least 21% of the lamb deaths observed on eight farms were attributable to ewe mortality [14]. Knowledge of the impact of ewe mortality on overall lamb mortality would help direct both further research and management interventions to improve lamb survival.

The objectives of this study were to (1) investigate the risk factors for, and causes of, lamb mortality in lambs born to three cohorts of ewe hoggets on two commercial farms in New Zealand; (2) evaluate the frequency and cause of hogget death over the lambing period and the impact of this on lamb loss rates; and (3) compare causes of mortality of lambs born to ewe hoggets and to mature-age ewes on the same farm when both age groups were bred and lambed at the same time.

## 2. Materials and Methods 

### 2.1. Lamb Mortality in Lambs Born to Ewe Hoggets (Objective 1)

#### 2.1.1. Farms and Animals 

Three cohorts of ewe hoggets from two commercial farms in the North Island of New Zealand were included in this study. Farm A was located in the Wairarapa region and comprised Romney breed sheep. Two cohorts of ewe hoggets from Farm A were included in the study and were born in the spring of 2016 and 2017, respectively, and thus were first bred and lambed in 2017 and 2018 (cohort A1 and A2, respectively). Farm B was located in the Waikato region and comprised Coopworth-cross sheep; one cohort of ewe hoggets born in the spring of 2015, thus bred and lambed in 2016, was included (cohort B). Ewe hoggets were individually identified using both an electronic identification tag and a plastic numbered tag that could be read from a distance. A total of 1142 ewe hoggets and 1348 lambs born to these ewe hoggets were included in the analysis. 

#### 2.1.2. Reproductive and Health Management 

In all cohorts, ewe hoggets were bred to entire rams at a ratio of 1:50–1:80 for 28–42 days. Pregnancy diagnosis was undertaken in mid-pregnancy using trans-abdominal ultrasonography to identify non-pregnant ewe hoggets and those carrying single or multiple (twin or triplet) fetuses. Non-pregnant ewe hoggets were removed from the study. For cohorts A1 and A2 all pregnant ewe hoggets that gave birth were included in the study (*n* = 509 and 241, respectively). On farm B, only those ewe hoggets that were bred in the first 10 days (based on ram harness crayon marks) and that were carrying single lambs were included in the study (*n* = 392).

In all cohorts, ewe hoggets were vaccinated against Toxoplasma gondii with a single dose of a modified live vaccine given greater than 4 weeks prior to the start of breeding (Toxovax^®^, MSD Animal Health, Wellington, New Zealand). They were also vaccinated against *Campylobacter fetus fetus* and *Campylobacter jejuni* with a sensitiser dose followed by a booster 4–6 weeks later, prior to breeding (Campyvax4^®^, MSD Animal Health, Wellington, New Zealand). Additionally, they were vaccinated against the *following Clostridial organisms: Cl. perfringens Type D, Cl. novyi, Cl. chauvoei, Cl. septicum, Cl. tetani* (Ultravac 5in1^®^, Zoetis, Auckland, New Zealand), receiving a sensitiser dose at weaning, a booster dose 4–6 weeks later and an additional booster dose 3–4 weeks prior to the planned start of lambing. From weaning through to mid-gestation, they received an effective broad-spectrum anthelmintic treatment at regular intervals. 

#### 2.1.3. Grazing Management 

Throughout gestation and lambing, ewe hoggets were grazed on permanent pasture which was predominantly comprised of ryegrass (*Lolium perenne*) and white clover (*Trifolium repens*). Grazing decisions were made by the farm managers under normal commercial conditions and no pasture measurements were taken. Seven to 15 days prior to the planned start of lambing, ewe hoggets were allocated to lambing paddocks at a rate of 6–8 per hectare (set-stocking) and remained in those paddocks until mid-lactation (tailing). 

#### 2.1.4. Weight and Body Condition Score (BCS) Data

The weight and BCS of each ewe hogget were collected at the start of breeding, end of breeding, mid-pregnancy (at the time of pregnancy diagnosis) and prior to lambing from all cohorts (Table 1). Ewe hoggets were weighed to the nearest 0.5 kg in a commercial weigh crate. Average daily gain (ADG) between time periods was calculated as the weight change between time points divided by the number of days and was expressed in grams per day. BCS was determined using a 5-point scale (1.0–5.0, in 0.5 intervals), indicating a score of 1.0 as emaciated and 5.0 as obese [15,16]. The BCS measurements were undertaken by a single experienced technician at all time points.

#### 2.1.5. Lambing Management and Lamb Necropsy

Starting 2–4 days prior to the planned start of lambing and continuing throughout the lambing period, ewe hoggets were monitored by researchers twice daily at approximately 8 am and 3 pm. Recently-born lambs, whether alive or dead, were ear-tagged and weighed. Their tag number, dam tag number, litter size, gender, and birthweight were recorded. Any lamb that required human assistance (correction of malpresentations and/or traction) to be born was recorded. Dead lambs were collected twice daily during the daily lambing monitoring. Dead lambs were either necropsied fresh (within 12 h of collection) or were frozen within four hours of collection and then later defrosted and necropsied within the following four weeks. 

Lamb necropsies were undertaken by an experienced veterinarian and cause of death was attributed to: (1) dystocia (Dystocia A characterized by subcutaneous oedema, presence or absence of walking and breathing, fat deposits not mobilized [9], or if they were recorded as requiring assistance at birth); (2) starvation-mismothering-exposure complex (SME; characterized by having breathed and walked, may or may not have fed, had mobilized most or all body fat stores [17]); (3) stillborn (characterized by not breathing and with no external evidence of dystocia nor recorded evidence of lambing assistance); (4) unknown (no obvious cause of death); (5) other (e.g., infection, congenital defect). No infectious disease testing was undertaken.

### 2.2. Ewe Hogget Mortality over the Lambing Period (Objective 2)

During the twice-daily monitoring over the lambing period, any ewe hogget that was found dead was recorded along with the likely cause of death if it was obvious (i.e., dystocia, vaginal or uterine prolapse, mastitis); if not obvious, the death was attributed to unknown causes. Live ewe hoggets with dystocia were assisted and recorded while those with vaginal or uterine prolapse were humanely euthanized and their tag number recorded. 

### 2.3. Comparison of Mortality in Lambs Born to Ewe Hoggets and Mature-Age Ewes (Objective 3)

On Farm A, at the start of breeding (Table 1) a cohort of mature-age (multiparous three and four-year old) Romney ewes was mixed with the A1 cohort of ewe lambs and bred to the same rams. At the end of breeding they were separated from the ewe hoggets and managed separately until weaning [4]. These mature-age ewes had been vaccinated against *Toxoplasma gondii*, *Campylobacter fetus fetus*, *Campylobacter jejuni*, and Clostridial organisms when they were ewe hoggets, as described in Section 2.1.1. They received a booster dose of Campyvax4^®^ (MSD Animal Health, Wellington, New Zealand) prior to breeding as two-tooth ewes (17 months of age). Each year, including the year of the study, they received a booster dose of Clostridial vaccine (Ultravac 5in1^®^, Zoetis, Auckland, New Zealand) three weeks prior to the planned start of lambing. At set-stocking they were allocated to lambing paddocks according to age, cycle of breeding, and number of fetuses they were carrying [4]; these were separate lambing paddocks from the A1 cohort of ewe hoggets but all lambing paddocks were in a similar area of the farm. During lambing, the mature-age ewes were monitored twice daily at the same times and in the same manner as that described for ewe hoggets in Section 2.1.4. Similarly, dead lambs were collected and necropsied as described in Section 2.1.4. 

All the procedures undertaken in the present study were approved by the Massey University Animal Ethics Committee (MUAEC 15/34 and 17/16).

### 2.4. Statistical Analyses 

Data were stored in Microsoft Excel^®^, while all statistical analyses were conducted using SAS (SAS Institute Inc., Cary, NC, USA, Version 9.4). 

#### 2.4.1. Causes and Risk Factors for Lamb Mortality for Lambs Born to Ewe Hoggets (Objective 1)

Lambs born to ewe hoggets were assigned a fate during the lambing period (Dead vs. Alive) based on the necropsy data. Thus, the outcome variable was lamb mortality during the lambing period, which was measured at the lamb level and was the unit of analysis. Predictor variables that were considered included: farm and year of lambing, lamb birthweight, total litter weight (i.e., total birth weight of the litter, if a singleton it was the weight of that singleton), birth rank (single vs. multiple born; triplets were included as multiple born as there were only 9 triplet-born lambs), sex of the lamb, ewe hogget liveweight (at start of breeding, pregnancy diagnosis, and pre-lambing), ewe hogget ADG (total average daily gain, including conceptus mass from start of breeding to pregnancy diagnosis, pregnancy diagnosis to pre-lambing, and start of breeding to pre-lambing), and ewe hogget BCS (at start of breeding, pregnancy diagnosis, and pre-lambing). Firstly, variables were examined using either *t*-test (continuous variables) or χ^2^ (categorical variables). Then, univariate generalized estimating equation models were used to examine the association between each predictor variable and the outcome variable. If variables were associated (*p* < 0.2), they were considered for inclusion in the initial multivariable models. Multivariable generalized estimating equation models, using an exchangeable correlation structure to account for clustering of lambs born to the same hogget (i.e., where there were multiple-born lambs), were developed. Forward manual variable selection was used to create the models, with variables retained where *p* < 0.05, after which the effect of adding variables in different orders was investigated (but without effect on the chosen model). Interaction terms that were biologically plausible were considered, and the interaction between lamb birthweight and birth rank was retained (*p* = 0.003). Continuous variables (e.g., liveweight) are presented in a graphical format, with predicted mean and 95% CIs. Categorical variables are presented as OR and 95% CIs. An analysis of stillborn lambs was undertaken in which the outcome variable was ‘Stillborn’ (Yes vs. No) based on the necropsy results, with lambs that survived the lambing period included in the ‘No’ category as well as those that died due to all other causes. The same predictor variables as above were considered, and the same process for univariate and multivariable models was also followed. No interactions were significant (*p* > 0.05) in the final model. 

#### 2.4.2. Ewe Hogget Mortality over the Lambing Period (Objective 2)

To evaluate frequency and cause of hogget death over the lambing period and its impact on lamb loss, descriptive data were collated and presented in table form.

#### 2.4.3. Comparison of Mortality in Lambs Born to Ewe Hoggets and Mature-Age Ewes (Objective 3)

To compare causes of mortality of lambs born to ewe hoggets compared with mature-age ewes, descriptive data were collated and presented in table form. Chi-square statistics were used to calculate differences between the age groups.

## 3. Results

### 3.1. Risk Factors for Lamb Mortality for Lambs Born to Ewe Hoggets (Objective 1)

#### 3.1.1. Descriptive and Univariate Analysis

Data from 1348 lambs born to 1142 ewe hoggets were included in the analysis. Overall, 78% of lambs were classified as alive by the end of the lambing period while 22% died during this time (Table 2). There were no differences in lamb mortality between cohorts (*p* = 0.09). 

Of the 1348 lambs that were born, 948 were singletons and 400 were multiple-born. Singleton lambs had lower odds of death compared with multiple-born (OR 0.59; 95% CI, 0.44–0.80; *p* < 0.0001). Singleton lambs had a predicted probability of mortality of 19.2% (95% CI, 16.8–21.8%), compared to 28.7% (95% CI, 23.9–34%) for multiple-born lambs. There was no difference between male and female lambs (*p* = 0.60).

Lambs with low birthweights were significantly more at risk of mortality (*p* < 0.0001). There was no association (*p* > 0.05) between lamb mortality and ewe hogget liveweight or BCS at any time point. The ADG of ewe hoggets during the period from the start of breeding to pre-lambing was significantly associated with lamb mortality, such that the higher the daily gain, the greater the risk of lamb mortality (*p* = 0.008).

#### 3.1.2. Multivariate Analysis

**Figure 1:**
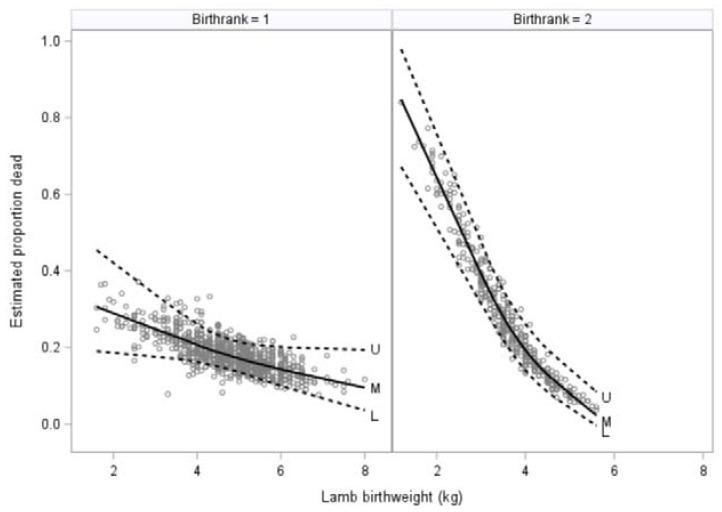
Estimated proportion of lamb deaths (*n* = 297) compared with lamb birthweight (kg) for 948 singleton lambs (Birthrank = 1; left) and 400 multiple-born lambs (Birthrank = 2; right) born to three cohorts of ewe hoggets on two farms. The solid black line shows the mean while the broken lines indicate 95% confidence intervals.

**Figure 2:**
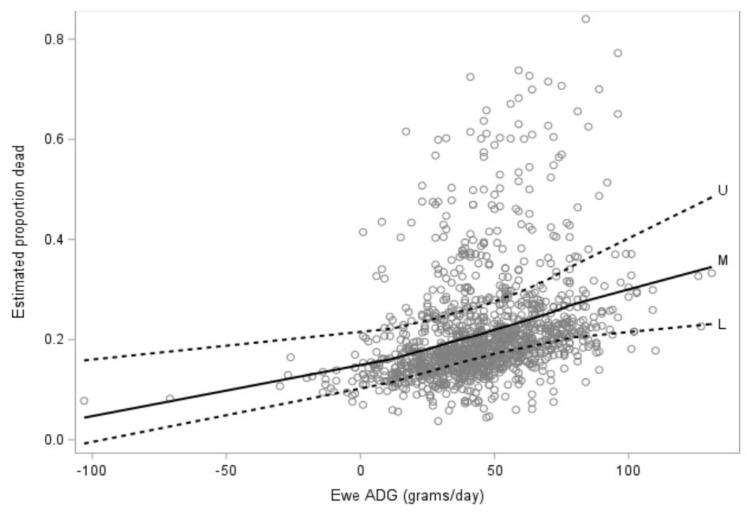
Estimated proportion of lamb mortality (*n* = 297) compared with ewe hogget average daily gain (ADG including conceptus mass, grams/day) between the start of breeding to pre-lambing (seven to 15 days prior to the planned start of lambing) for 1142 ewe hoggets. The solid black line shows the mean while the broken lines indicate 95% confidence intervals.

The final multivariate model evaluating whether lambs were alive or dead in the lambing period included lamb birthweight (*p* < 0.0001) which was seen for both singleton and multiple-born lambs although was more marked for the latter (Figure 1), lamb litter size (*p* = 0.0003), ewe hogget ADG from start of breeding to pre-lambing (Figure 2; *p* = 0.018), and the interaction between lamb birthweight and lamb litter size (*p* = 0.003). 

### 3.2. Causes of Lamb Mortality for Lambs Born to Ewe Hoggets (Objective 1)

#### 3.2.1. Descriptive and Univariate Analysis

Necropsies were performed on 297 lambs that died in the lambing period. In all three cohorts the main cause of death was attributed to stillborn, with a total of 41% of deaths attributed to this cause (Table 3). There were no significant differences between cohorts in the percentage of dead lambs that were stillborn (*p* = 0.21). 

Stillborn lambs had lower birthweights (*p* < 0.0001) than lambs that were not stillborn (Table 4). Singleton lambs had lower odds of being stillborn compared with multiple-born lambs (OR 0.58; 95% CI, 0.38–0.87; *p* = 0.008). There was no difference in stillbirth between male and female lambs (*p* = 0.14).

There was no association with the odds of being stillborn and ewe hogget liveweight or BCS at any time point (*p* > 0.05). The ADG of ewe hoggets from start of breeding to pre-lambing was significantly associated with lambs being stillborn, such that the higher the daily gain of the ewe hogget, the greater the risk of their lamb(s) being stillborn (*p* = 0.008).

#### 3.2.2. Multivariate Analysis

**Figure 3:**
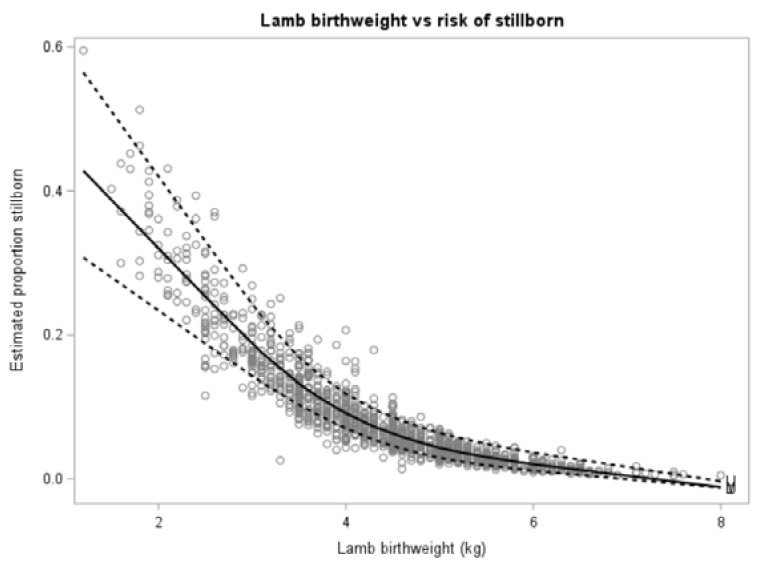
Estimated proportion of lamb mortality due to stillborn (*n* = 122) compared with lamb birthweight (kg) for 1348 lambs born to three cohorts of ewe hoggets on two farms. The solid black line shows the mean while the broken lines indicate 95% confidence intervals.

**Figure 4:**
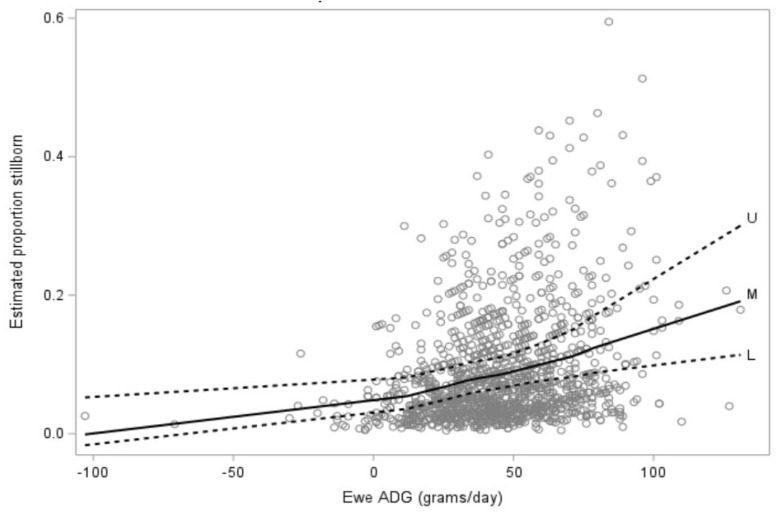
Estimated proportion of lamb mortality due to stillborn (*n* = 122) compared with ewe hogget average total daily gain (ADG including conceptus mass, grams/day) between the start of breeding to pre-lambing (seven to 15 days prior to the planned start of lambing) for 1142 ewe hoggets. The solid black line shows the mean while the broken lines indicate 95% confidence intervals.

In the multivariable model for risk of stillbirth, lamb birthweight (*p* < 0.0001; Figure 3) and ewe hogget ADG from start of breeding to pre-lambing (*p* = 0.007; Figure 4) were included. No other variables or interactions were significant or improved model fit. 

### 3.3. Ewe Hogget Mortality over the Lambing Period (Objective 2)

During the lambing period, 29 of the 1142 (2.5%) ewe hoggets in the three cohorts died or were euthanised, with the most common causes of death being dystocia followed by vaginal prolapse (Table 5). These 29 ewe hogget deaths resulted in 33 lamb deaths, accounting for approximately 11% of the total lamb deaths in the study. An additional 20 hoggets (2, 12, and 6 from cohorts A1, A2, and B, respectively; 1.8% of the total ewe hoggets) had an assisted lambing but survived; it is unknown if they would have died or survived if assistance had not been provided.

### 3.4. Comparison of Lamb Mortality for Lambs Born to Ewe Hoggets and Mature-Age Ewes (Objective 3)

Necropsy data from 273 dead lambs from a total of 1979 lambs born to 1050 mature-age ewes from Farm A in 2017 were available for analysis. The most commonly attributed cause of lamb mortality for lambs born to ewe hoggets in cohort A1 was stillborn (46%) whereas for lambs born to mature-age ewes at the same time on the same farm only 7% of deaths were attributed to stillborn (*p* < 0.001; Table 6). Lambs born to ewe hoggets were less likely to die from unknown or other causes (*p* < 0.001) compared to those born to mature-age ewes. There were no differences between the ewe age groups in the likelihood of lambs dying from dystocia or SME (*p* > 0.05). 

## 4. Discussion

In this study, 78% of lambs survived through the lambing period. This is a similar lamb survival rate to that reported in other studies with ewe hoggets where survival to marking or weaning ranged from 61–89% [2,5,6,7,8]. These studies emphasise the potential for large lamb losses in ewe hoggets, higher than what is typically reported for mature-age ewes. Low lamb birthweight was an important risk factor for ewe hogget lamb mortality in this study, contributing to both the risk of mortality generally and the risk of being stillborn. Multiple-born lambs were also at higher risk of death than singletons, both overall and from stillborn. Other authors have reported that lamb mortality in lambs born to mature-age ewes also increases at higher birth weights [10,11], but this was not seen in the present study. It is not clear why this is, although it is possible that there were relatively fewer high birthweight lambs compared to other studies, which used mature-age ewes. The mean lamb birthweights in the present study were comparable to those reported in other studies using similar-breed lambs from ewe hoggets managed in a similar way [2,5,18,19].

It is well-established that lambs with low birthweights have poor vigour, udder-seeking behaviour, and thermo-regulatory ability [20] and therefore management strategies to increase the birthweight of lambs born to ewe hoggets, particularly for multiple lambs, should be investigated. For example, mid-pregnancy shearing has been shown to sometimes result in an increase in lamb birthweight (reviewed by [21]), although most of those studies were undertaken in mature-age ewes with little effect seen in young age ewes (reviewed by [1]). Lambs born to ewe hoggets with higher genetic merit for post-weaning weight have been reported to have higher birthweights [22], as have lambs born to ewe hoggets that were heavier and older at breeding [8]. Improved maternal nutrition during pregnancy has been reported to improve lamb survival [12,13] and birthweight [13]. However, studies of differential feeding of groups of ewe hoggets during gestation under commercial pastoral conditions showed minimal or no impacts on lamb birth weight and survival based on group means (reviewed by [1]), indicating that increased nutrition of groups of ewe hoggets during pregnancy may not increase birthweight and/or survival. Indeed, in the present study, higher average daily gain during gestation was associated with reduced lamb survival. However, good nutritional status of ewe hoggets during gestation is still essential to developing an adequate frame-size to reduce the risk of dystocia due to fetopelvic disproportion [9] and ensure they are well-grown for re-breeding as two-tooth ewes [1]. Stillborn was the most commonly recorded cause of lamb death in all cohorts of ewe hoggets, accounting for 31–46% of recorded lamb deaths. In cohort A1, the percentage of stillborn lambs born to ewe hoggets was significantly greater than in those born to mature-age ewes that were lambing at the same time. The rates of stillborn in lambs born to ewe hoggets in the present study were also higher than the 15% reported in a recent Australian study [7]. Stillborn lambs had lower birthweights than non-stillborn lambs, which raises the possibility that some may have been born prematurely, but as conception dates were not known for the ewe hoggets it was not possible to investigate gestation length. For future studies investigating the mortality of lambs born to ewe hoggets, it is recommended that an assessment of gestation length is included in the study design. However, another outcome from this study was that increasing average daily liveweight gain of ewe hoggets from the start of breeding to pre-lambing (i.e., during gestation) was associated with an increased risk of lamb mortality and risk of lambs being stillborn. This finding was consistent in all three cohorts and was unexpected, as it is the opposite of what was found in a New Zealand study investigating the likelihood of ewe hoggets rearing a lamb to weaning [6]. However, in that study, udder palpation of the ewe hoggets at weaning, to determine whether or not they were actively lactating, was used as a proxy for lamb survival, so it was not possible to determine lamb birthweights or the cause or timing of deaths [6]. It should also be noted that in the present study, relatively few ewe hoggets lost weight during pregnancy compared with [6]. During gestation, ewe hoggets are themselves still growing. The finding that lamb mortality was greater in lambs born to more rapidly growing ewe hoggets raises the possibility of whether some ewe hoggets preferentially partitioned nutrients towards their own growth, rather than towards placental and fetal development, resulting in lower birthweight lambs with poorer survival. This has been demonstrated in housed ewe hoggets fed to achieve very high daily weight gain (reviewed by [23]), although the weight gains achieved in those studies were substantially greater than those of the present study and are unlikely to occur in pregnant ewe hoggets grazing on pasture [1]. 

Different authors have used varying methodology and case definitions to attribute causes of perinatal lamb mortality, particularly for dystocia and/or stillborn. While the presence of vascular congestion or haemorrhage of the superficial meninges and spinal cord has been used in the diagnosis of dystocia [24], there may be some inaccuracy in this methodology [17,25] and interpretation of these lesions may vary depending on the experience and biases of the operator [9]. In the present study, the presence or absence of vascular congestion or haemorrhage of the superficial meninges and spinal cord were not evaluated as part of the lamb necropsy process, due both to the potential inconsistencies in this as a diagnostic tool and because a number of the lambs had been frozen prior to necropsy which may affect the appearance of the blood vessels in the central nervous system [9]. Therefore, lamb deaths were only attributed to dystocia if there was presence of subcutaneous oedema (Dystocia A; reviewed by [9]) or if the ewe required human assistance to give birth. It is therefore possible that some of the lamb mortalities attributed to stillborn in this study may have suffered dystocia, which may have affected their viability.

Infectious diseases such as *Toxoplasma gondii* and *Campylobacter fetus fetus* may potentially result in stillbirth, however all ewe hoggets in the present study were fully vaccinated against these diseases. *Chlamydia pecorum* has been reported in stillborn lambs in Australia [7] and has also been reported in aborted fetuses in New Zealand [26], but in the present study no infectious disease testing was undertaken. New Zealand is free from other known diseases that potentially result in stillbirth such as *Chlamydia abortus* and *Coxiella burnetiid* [27]. In future studies of mortality of lambs born to ewe hoggets, investigation of potential infectious disease in stillborn lambs should be considered. Regardless of cause, if high rates of stillborn lambs also occur in other flocks of lambing ewe hoggets then it represents a significant cause of lamb wastage and further research is indicated to investigate the causes of stillborn.

Overall, 2.5% of ewe hoggets died during the lambing period, accounting for approximately 11% of total lamb mortalities, emphasising the impact that dam deaths can have on lamb wastage. This hogget mortality rate during the lambing period is similar to that reported in a study of two- and three-year-old ewes [28]. The most common cause of ewe hogget death in the present study was dystocia, resulting in the death of 0.9% of the ewe hoggets while an additional 1.8% were given assistance at lambing but survived. Assuming that most or all of these would have died without assistance, these results demonstrate that there is value in monitoring ewe hoggets during the lambing period to identify and correct dystocia. The prevalence of vaginal prolapse has not previously been reported in ewe hoggets in New Zealand; in the present study it was 0.7%, which is similar to the 1% mean prevalence reported in mature-age ewes in New Zealand [29].

## Figures and Tables

**Table 1:** Schedule of management events and data collection time points for a study investigating ewe hogget and lamb mortality during the lambing period in three cohorts from two farms.

	Cohort		
Event	A1	A2	B
Start of breeding	7 May 2017 ^1^	5 May 2018 ^1^	11 April 2016 ^1^
End of breeding	19 June 2017	8 June 2018	29 April 2016
Pregnancy diagnosis (mid-pregnancy)	8 August 2017 ^1^	6 August 2018 ^1^	15 July 2016 ^1^
Set-stocking (pre-lambing)	23 September 2017 ^1^	25 September 2018 ^1^	23 August 2016 ^1^
Start of lambing	4 October 2017	2 October 2018	8 September 2016
End of lambing	16 November 2017	5 November 2018	23 September 2016

^1^ Weight and body condition score data collected.

**Table 2:** Number (and percentage) of lambs born to 1142 ewe hoggets that were alive or died during the lambing period from three cohorts on two farms.

	Cohort			
Status	A1	A2	B	TOTAL
Alive	530 (80%)	230 (78%)	291 (74%)	1051 (78%)
Dead	132 (20%)	64 (22%)	101 (26%)	297 (22%)
TOTAL	662	294	392	1348

**Table 3:** Number (and percentage) of lamb mortalities attributed to various causes in 297 lambs born to three cohorts of ewe hoggets on two farms.

	Cohort			
Cause of Mortality	A1	A2	B	TOTAL
Dystocia	13 (9.8%)	19 (29.7%)	18 (17.8%)	50 (16.8%)
Starvation Mismothering Exposure complex	49 (37.1%)	14 (21.9%)	14 (13.9%)	77 (25.9%)
Stillborn	60 (45.5%)	20 (31.3%)	42 (41.6%)	122 (41.1%)
Unknown	7 (5.0%)	8 (12.5%)	18 (17.8%)	33 (11.1%)
Other	3 (2.3%)	3 (4.7%)	9 (8.9%)	15 (5.1%)
TOTAL	132	64	101	297

**Table 4:** Mean (and Standard Error of Mean) lamb birthweights, differentiated by gender, litter size, and whether or not they were stillborn, from 1348 lambs born to three cohorts of ewe hoggets on two farms.

	Cohort			
Lamb Type	A1	A2	B	OVERALL
All lambs	4.16 (0.04)	4.52 (0.06)	4.78 (0.05)	4.42 (0.03)
Female	4.08 (0.05)	4.25 (0.08)	4.61 (0.06)	4.27 (0.04)
Male	4.24 (0.06)	4.80 (0.09)	4.96 (0.08)	4.57 (0.05)
Single-born	4.64 (0.05)	4.98 (0.07)	4.78 (0.05)	4.76 (0.03)
Multiple-born	3.58 (0.05)	3.72 (0.07)	N/A	3.62 (0.04)
Not stillborn	4.25 (0.04)	4.61 (0.06)	4.84 (0.05)	4.50 (0.03)
Stillborn	3.19 (0.17)	3.26 (0.26)	4.28 (0.18)	3.57 (0.12)

**Table 5:** Number (and percentage of total) of ewe hoggets that died from various causes during the lambing period in 1142 ewe hoggets from three cohorts on two farms.

	Cohort			
Cause of Mortality	A1 *n* = 509	A2 *n* = 241	B *n* = 392	Total *n* = 1142
Dystocia	4 (0.8%)	1 (0.4%)	5 (1.3%)	10 (0.9%)
Vaginal prolapse	3 (0.6%)	1 (0.4%)	4 (1.0%)	8 (0.7%)
Uterine prolapse	0 (0.0%)	0 (0.0%)	6 (1.5%)	6 (0.5%)
Unknown	1 (0.2%)	1 (0.4%)	1 (0.3%)	3 (0.5%)
Other	0 (0.0%)	2 (0.8%)	0 (0.0%)	2 (0.2%)
Total	8 (1.6%)	5 (2.1%)	16 (4.1%)	29 (2.5%)

**Table 6:** Number (and percentage) of lamb mortalities attributed to various causes in 132 lambs born to ewe hoggets and 273 lambs born to mature-age ewes from the same flock that were bred and lambed at the same time.

	Cohort	
Cause of Mortality	A1 Ewe Hoggets	Farm A Mature-Age Ewes
Dystocia	13 (9.8%)	45 (16.5%)
SME	49 (37%)	117 (42.9%)
Stillborn	60 (45.5%) *	18 (6.6%) *
Unknown	7 (5.0%) *	56 (20.5%) *
Other	3 (2.3%) *	37 (13.5%) *
Total	132	273

* Significant difference between ewe age groups, *p* < 0.001.

## Data Availability

The data utilised in this study are available on request from the corresponding author.
